# Facilitators and barriers to birth preparedness and complication readiness in rural Rwanda among community health workers and community members: a qualitative study

**DOI:** 10.1186/s40748-018-0080-6

**Published:** 2018-06-06

**Authors:** Richard Kalisa, Patrick Smeele, Marianne van Elteren, Thomas van den Akker, Jos van Roosmalen

**Affiliations:** 1 Department of Obstetrics and Gynecology, Ruhengeri Hospital, Musanze, Rwanda; 2 Athena Institute, VU University, Amsterdam, The Netherlands; 3 Department of Medical Humanities, VU University Medical Center, Amsterdam, The Netherlands; 4 Department of Obstetrics, Leiden University Medical Center, Leiden, The Netherlands

**Keywords:** Pregnancy, facility delivery, birth preparedness, Perceptions, Respectful maternity care

## Abstract

**Background:**

Birth preparedness and complication readiness (BP/CR) comprise a strategy to make women plan for birth and encourage them to seek professional care in order to reduce poor pregnancy outcome. We aimed to understand the facilitators and barriers to BP/CR among community health workers (CHWs) and community members in rural Rwanda.

**Methods:**

Eight focus group discussions were conducted with 88 participants comprising of CHWs, elderly women aged 45–68 and men aged 18–59, as well as two key informant interviews in Musanze district, Rwanda, between November and December 2015. Qualitative data were digitally recorded, transcribed verbatim and analysed using content analysis.

**Results:**

Participants perceived the importance of family assistance, medical insurance and attending antenatal care (ANC) to facilitate BP/CR and enhance professional care at birth. CHWs reinforced BP/CR messages by SMS alerts and during community gatherings. ‘Ubudehe (collective action to combat poverty)’ was known as a tool to identify the poorest families in need of government aid to pay for medical care. Disrespect and abuse of women during labor by health workers were perceived as important barriers to access professional care, as well as conflicting health policies such as user fees for ANC and family planning services, and imposing fines on women giving birth outside health facilities.

**Conclusion:**

CHWs, ANC and medical insurance are perceived to be important facilitators of BP/CR. Respectful care is paramount for improved maternal health. There is a need for addressing inconsistent health policies hindering the intention to access professional care.

## Background

Access to maternal and newborn health services may be hampered by cultural, socioeconomic, and demographic factors [1]. Receiving care from skilled birth attendants [2–4] and birth preparedness and complication readiness (BP/CR) to encourage such attendance of professional care continue to be key components in programs to reach the Sustainable Development Goals (SDGs) for improved maternal and neonatal survival [5, 6].

BP/CR was previously shown to promote active preparation and decision making for birth among pregnant women and their families [2, 6–8]. Recommendations for birth planning include identifying a birth attendant and the closest appropriate care facility; saving funds for birth and emergency expenses; making arrangements for transport; and looking for compatible blood donors [5].

In 2003, Rwanda adopted BP/CR as part of focused antenatal care to increase facility births [9]. This may have contributed to the reduction of the maternal mortality ratio by roughly two-thirds from 750 in 2005 to 210 per 100,000 live births in 2015 and an increase in skilled birth attendance rate from 28% to 91% in this same time frame [10, 11]. This study aimed to understand the facilitators and barriers to BP/CR among community health workers (CHWs) and community members in rural Rwanda.

## Methods

We used a qualitative content analysis [12] to explore perceptions held by CHWs and community members’ about BP/CR. The study was conducted in Musanze district, northern Rwanda, with a population of 368,267 inhabitants and a skilled birth attendance rate of 66% [11]. In addition, there is access to one provincial referral hospital for high-risk obstetric cases from 12 health centers and other district hospitals in the northern province. It is also supported by CHWs and a SMS-based system which was developed to improve maternal and child health (MCH) using RapidSMS® that enables communication between CHWs at community level, and the rest of the health system (ambulance, health facility staff, district hospital and RapidSMS-MCH system) through mobile phones [13]. The CHWs referred to throughout this paper were responsible for MCH activities and covered a catchment area of 100–150 households. Medical services offered are paid for using community-based health insurance (commonly known as mutuelles de santé), with an annual fee of RWF 3000 (US$ 4), plus a 10% co-payment for each episode of illness.

### Sampling and recruitment procedures

Different sampling strategies were used for different groups of participants. We applied purposeful sampling to recruit CHWs, elderly women aged 45–68 and men aged 18–59. First, we used the existing list of all active CHWs covering the entire district and then randomly selected four CHWs to represent one health centre (HC), and continued the same process to enrol other CHWs from the remaining eleven HCs. The selected representative CHWs from one HC were joined with two additional groups of CHWs from other HCs to make up one focus group discussion (FGD), which was held at an agreed nearby location where privacy was ensured. While elderly women and men were selected with assistance from CHWs after introducing the aim of the study to them. CHWs were asked to select those elderly women who had daughters or daughters-in-law and men who had a female partner, daughters or daughters-in-law, who delivered within the last two years assuming that these elderly women and men had some knowledge of BP/CR.

### Data collection

Data collection took place in November and December 2015 using pretested FGDs and key informant interviews (KIIs) guides which were developed in English and translated in Kinyarwanda. Topics covered in the discussion guide were: steps taken to prepare for birth and potential complications during birth in the community, cultural and social issues surrounding BP/CR, recognition of maternal and newborn danger signs, access towards skilled care and challenges at community level. We conducted a total of 8 focus group discussions, with a total of 88 participants from the following groups: CHWs (four groups), elderly women (two groups) and men (two groups). Each discussion included 8 to 12 members. We used two KIIs based on their position to inform policy with regards to maternal health within the district; one with the nurse-in-charge from the district hospital and the head of CHWs at district level respectively. Two other participants for the planned KIIs refused to take part after several attempts to meet them for interview.

The interviews were carried out in Kinyarwanda, the national language. In FGDs, we encouraged all participants to contribute and each session took approximately 60 to 90 min. Interviewers briefed participants on study aims and that the conversation would be audio-recorded and verbal consent obtained at the beginning of the FGDs and KIIs. One researcher with experience in conducting qualitative studies (RK) who is fluent in the local language assisted by second author (moderator/ note taker) conducted all the FGDs and KIIs. The KIIs were held at the district hospital. All participants received Rwandan Francs 4000 (US$ 5) to compensate for their time, which was consistent with the standard payment dispensed by nongovernmental organizations active in the sites.

### Data analysis

All FGDs and KIIs were transcribed verbatim in Kinyarwanda and then translated into English by the first author and a research assistant increasing their familiarity with the data. The transcripts were then entered into the NVivo 10 software package to aid in the coding of the data and analysis was then conducted using qualitative content analysis [12, 14]. The first author and a qualitative research assistant independent read and reread the transcripts, and coded the transcripts line by line to identify meaning units. Similar meaning units were grouped into condensed meaning units. Further coding was performed to expound on the condensed meaning units and then grouped together through an inductive and deductive process to develop categories. Categories were further checked with the data to ensure they reflect the content of the transcripts and constant comparison done between categories to develop the final themes.

## Results

During the analysis, two categories emerged from the FGDs and KIIs, related to BP/CR, which we labelled ‘Pathway’ and ‘Modifying factors’, with each subdivided into several subcategories. Quotes are given to illuminate views of participants.

### Pathway

#### Financial savings

Families tried to save money during pregnancy to ensure access to labor supplies, transport and medical insurance prior to labor.

*“During our home visits to pregnant women we do motivate men to provide financial support for their wives... However, in cases of unwanted or teenage pregnancies from poor families this is not always the case. So you find women struggle on their own and for those who cannot afford it* (*insurance*), it *becomes a challenge to access medical care so they give birth at home.”* (CHWs FGD 1).

#### Linkage to care

CHWs identify pregnant women and were often the first to be consulted for questions with regard to pregnancy. CHWs were seen to provide advise with regard to HIV testing and counseling, birth planning, personal hygiene and medical insurance, vaccines and nutritional supplements.

*“After you have been told that there is a pregnant woman in your village, you visit her at their home where you will inquire if it’s true she is pregnant. And if she isn’t sure then escort her to the health center for pregnancy test first. After it’s confirmed, I will register her in my book and send a Rapid SMS to Ministry of Health system (Rapid SMS-MCH System) which continues to send me reminders about her next ANC visit…”* (CHW FGD 3).

CHWs provide postpartum care including surveillance of severe vaginal bleeding, abdominal pain and dizziness/anemia. Newborns are checked for abnormal body temperature, heart rate, weight, discharge and yellowness until one month of age.

*“They (CHWs) will visit the mother-baby pair to verify if both are in good health which will be followed by sending a Rapid SMS system confirming that, then continue with consecutive postnatal checkups on 3* ^*rd*^ *, 7* ^*th*^ *and 28* ^*th*^ *days” (KIIs; In-charge CHW at District level).*

#### Social/emotional support

Participants reported it was the responsibility of family members to support pregnant women in house chores such as cooking, washing clothes, fetching water and firewood collection. At community level, ‘family meetings’ were organized by village chiefs in order to create a dialogue as to how to solve socio-economic problems in the community. These meetings are known as ‘umuganda (gathering of the people of each neighborhood for community work once a month)’ or ‘umugoroba w'ababyeyi (parents’ evening forums)’ in which parents at the village level meet to discuss financial security, health and social issues, including intimate partner violence among pregnant women.

“*During these meetings, he (village chief) calls for community members who discuss on to why the family has delayed to pay their medical insurance contributions. If they find them (family) to lie within ‘poorest group’ using ubudehe* (collective action to combat poverty) *categories then a letter will be written to the sector administrator who communicates to the social affairs at district level…While those families categorized above ‘poorest’ they confiscate their property until they have paid..*.” (FGD 4 CHW).

### Modifying factors

#### Social factors

Participants reported that some couples refused to seek ANC services out of fear to be chased away for not coming with their spouses, since this is a requirement for being tested for HIV. Teenagers and single women were said to hide their pregnancies even up to term, avoiding to be asked about the father of the baby during ANC visits or labor. Others were ashamed to attend in old clothes or for not having baby layettes to show at the clinic. Multiparous women felt embarrassed to seek health care for being ‘laughed at’ as ‘family planning failures’.

*“I think too the requirement of a man attending ANC should be stopped. A woman might need to go for ANC when the husband is not around. If you take my case as an example, I work from upcountry and it is that job I make a living out of…, may want to come but my employer refuses to give me permission...Meaning my wife may miss out completely on her appointments.”* (Men FGDs 1).

Women perceived hospital costs as prohibitively high, especially for those without medical insurance barring unintended consequences to those women from poor backgrounds. Single or teenage women in most cases were not in stable relationships and not financially supported. Participants highlighted reasons for delayed enrolment into medical insurance programs, such as the need for everyone in the family to sign up and pay immediately upon enrolment or while other women enrolled towards term not knowing the insurance sometimes takes a month or longer to be functional.

*“… when labor starts, they will first attempt to be delivered at home and after failing come to the health center thus exposing them to severe obstetric complications. Nevertheless, we have done a lot of sensitization either through media or during CHWs home visits on the importance of health insurance which many families have adhered too…” (KII; Nurse-in-charge at district hospital).*

#### Structural factors

Reasons cited for women not using health facilities include difficult terrain hindering access, rapid progress of labor and most health centers did not have ready available ambulance to transport women during emergencies.

*“...we were escorting a woman to give birth at the health center, she reached a point and couldn’t walk anymore; so she squat and delivered. We still had 2 Km to reach the health center, so I called at the health center to send us an ambulance only to be told it had been sent elsewhere. Then requested that same midwife to come and assist me to cut the baby’s cord, instead she replied me to come and collect the kits myself... Finally, what I did was to organize with the local traditional stretcher ‘ingobyi’ team from the area she had delivered from and they assisted me to carry her...By the time we reached, baby appeared white and very cold but thank God they both survived”.* (CHWs FGD 2).

Participants expressed abandonment by health workers, dirty maternity units and rudeness of healthcare staff, particularly the midwives. When women go to a health facility they should be treated like human beings, with respect and not shouted at as they are brought in.

*“…some tell of sad stories about their deliveries and they even think that they should not give birth anymore because of the abuse and mistreatment that they receive.* (Men FGDs 1) *I remember way back when I had gone to deliver my baby instead of being assisted, the nurses kept insulting me (you enjoyed doing it, why are you screaming now), don’t try and scream here. Nurses are just there not helping; you wonder if it’s a health facility you were brought to?”* (Elderly women FGDs 2).

#### Inconsistent health policies factors

Most participants were supportive of women giving birth at the health facility, but they questioned inconsistent health policies regarding fee charges upon each ANC or family planning visit, which had previously been free. Also, fines imposed on women who had given birth *en* route or at home were perceived to be counterproductive.

“*Since the introduction of user fees upon each ANC visit, we are most likely to go back to those old days when women delivered at home. Even I told the in-charge of CHWs at district level that if nothing is done, our job as CHWs is coming to an end because women have started lying to us even their last menstrual period so that they can go for ANC once (towards term) in order to save money from the prior ANC visits to be able to buy baby clothes. Therefore, we propose that these fees should be removed so that women can attend ANC freely as it was previously.*” (CHWs FGD group 3).

## Discussion

Participants perceived the importance of family assistance, medical insurance and attending ANC, to facilitate BP/CR and enhance professional care at birth. Community health workers reinforced BP/CR messages by SMS alerts and during community gatherings. Disrespect and abuse of women during labor by health workers were perceived as important barriers to access professional care, as well as conflicting health policies such as user fees for ANC and family planning services, and imposing fines on women giving birth outside health facilities.

It has been well documented that in sub-Saharan Africa, as in other settings, BP/CR entails saving money for buying birth items and transport to health facilities [7, 8, 15]. In Rwanda, this was possible through CHW activities where they engaged with women/families on a one-to-one basis prior to delivery about birth preparedness [16]. In addition, community members saved money to comply with medical insurance but costs were still a barrier and even some challenged the threats associated with failure to comply with the guidelines and regulations used to pay premiums for medical insurance [17].

Our findings are consistent with other studies were CHWs aided skilled birth attendance [16, 18, 19]. In addition, they were the final decision makers in case of emergency obstetric care contrary to what other studies have reported that approval was needed first directly or indirectly from the husband or a family member respectively [20, 21]. This could be explained by a high level of trust and respect expectant mothers from rural Rwanda have for CHWs, whom they called on their mobile phones to offer assistance thus reducing delays in seeking care [13, 16].

This study has demonstrated how integration of mobile phone technology ensured that mother or neonate were timely transferred for emergency obstetric and neonatal care [13]. In addition, it has been suggested to be used as a continuing professional development tool to enhance clinical decision making in emergency obstetric care in low-resource settings [22, 23]. Other strategies were use of community gatherings ‘*umuganda*’ and ‘*umugoroba w’ababyeyi’* as a platform where issues and plans are discussed inclusive about the importance of BP/CR [17]. While *ubudehe* categorization identified families from poorest communities who benefited from free health care paid for by either government or development partners [24].

Our study has found barriers to utilization of ANC services such as fear of being tested for HIV, contrary to other authors [25, 26], women being chased away and asked to return with their partner to access care similarly to another study from urban Rwanda, but in that particular study women opted to pay a man who is not necessarily the partner – in order to access care [27]. Others were ashamed to attend in old clothes or for not having baby layettes to show at the clinic [8], and teenage/single mothers who hid their pregnancies even up to term after being rejected by the baby’s father, making them to go live with their mothers whom in most cases due to poor socio-economic status were more likely to face complex decision-making patterns [28]. Special attention must be paid to improve the interactions with this group and with their mothers as decision-makers.

Most studies have indicated that distance to health facility, rapid progress of labor and lack of preparation for transport hindered health seeking behavior and access to care, hence resulting in pregnant women deliver en route or at home [8, 28, 29]. Therefore, preventive strategies are of utmost importance, such as establishment of maternity waiting homes [30] in communities where it is difficult to access emergency obstetric care, when required.

Studies have, similarly to ours, reported abandonment and rudeness by midwives to women when they come to health facilities for delivery. The latter were described as unwelcoming and negatively affected the integrity of women seeking obstetric emergency care [31, 32]. Thus, a need exists to improve communication to make services appealing and trustworthy to women and their social networks [33] and recognize continuous support for respectful maternity care especially in our labor rooms [31–33].

Delivery in health facilities was reported to be mandatory. Our findings, however, reported inconsistent health policies which were perceived to be conflicting with BP/CR in a community which still reveals socio-cultural barriers towards women’s access to ANC and contraception services [34, 35], posing threats and should be addressed. In addition, caution should therefore be taken when introducing any financing reforms to ensure that the needs of the poor and vulnerable are protected and that they are implemented within the context of universal coverage [36].

The strength of this study is that we used both FGDs and KIIs to explore community perceptions of how BP/CR is discussed and used. In addition, we included different participants in terms of age, and gender because they provided different perspectives related to the topic of discussion. The limitation of our study is the social desirability bias rather than a genuine reflection. This could have occurred because the moderators and note-takers were of a medical background, and hence participants may have had a desire to show preference for the use of skilled care. In addition, the moderators were all male and this could have caused women to be shy and reserved. However, we believe this did not happen, as the participants were all vibrant and participated in dynamic discussions.

## Conclusions

In this community, perceptions regarding BP/CR were in favour of skilled care. Community health workers reinforced BP/CR messages by SMS alerts and during community gatherings (*umuganda or umugoroba w’ababyeyi*). Disrespect and abuse of women mentioned during labor by midwives together with some health policies such as user fees for ANC and family planning services, and imposing fines on women giving birth outside health facilities were perceived as important barriers to access professional care.

There is a need for addressing inconsistent health policies hindering the intention to access professional care. Additionally, to continue emergency preparedness in education to women and their spouses during ANC, key family members—mothers, mother in laws as well as other women in the community to address some barriers to making preparations or delivering in health facilities. We recommend further studies to understand more dimensions regarding disrespectful maternity care in Rwanda.

## Abbreviations

ANC: Antenatal Care
BP: Birth Preparedness
CHWs: Community Health Workers
CR: Complication Readiness
FGDs: Focus Group Discussions
HCs: Health centers
KIIs: Key informant interviews
NGO: Non-governmental Organization
SBA: Skilled Birth Attendant
